# The Complex and Multifactorial Nature of Alzheimer’s Disease

**DOI:** 10.2174/157015911798376235

**Published:** 2011-12

**Authors:** Karim Alkadhi, Jason Eriksen

In 1907 Alois Alzheimer, a Bavarian psychiatrist, published a seminal two-page article, entitled “A Characteristic Disease of the Cerebral Cortex”[1], followed by the publication of his second article “On Certain Peculiar Diseases of Old Age”, that detailed the clinical, biographical, and neuropathological history of two patients admitted under his care for pre-senile dementia [2]. In these reports, Alzheimer described these patients as suffering from a “state of profound mental impairment with prominent agnostic, aphasic, and apractic disturbances,” which he brilliantly attributed to small “miliary foci” and/or dense “bundles of fibrils” [1,2]. Even after a century of research, we continue to use the association between cognitive decline and proteinaceous deposits as classic hallmarks of Alzheimer’s disease.

With the spectacular advancements in medicine and technology in recent decades, life expectancy has significantly increased, and diseases once deemed untreatable can now be prevented or even cured. Nevertheless, even with the progress that has been made in improving human health, this shift in demographics has been accompanied by the increased appearance of chronic diseases associated with ageing. Among such disorders, Alzheimer’s disease remains one of the most feared, as it is an irreversible, largely age-related neurodegenerative brain disorder that is responsible for the gradual and insidious failure of cognitive function. Ageing is one of the most well-established risk factors for Alzheimer’s disease [3]; this disorder has emerged as the predominant form of dementia in the elderly, affecting one person in ten over the age of 65, and 50 percent of individuals over the age of 85 [4,5].

Although the specific causes of idiopathic Alzheimer’s disease remain unknown, the vast majority of genetic and pathological observations made over that the past two decades suggests that an initial and critical accumulation of amyloid-beta (Aβ) is a key initiating factor in the pathogenesis of this disease [6], leading to a variety of biochemical changes that include neuroinflammation, synaptic dyfunction, and tauopathy, eventually resulting in cell death, a process that has been dubbed the “amyloid cascade hypothesis.” Nevertheless, in the earliest stages of Alzheimer’s disease, β-amyloidosis correlates poorly with the degree of cognitive decline, implying that other of factors may contribute to AD progression. Recent reports show that as many as 30% of individuals older than age 75 who are considered clinically normal at the time of death, have neuropathological hallmarks of AD when autopsied. Despite the substantial AD lesions in these individuals, a lack of apparent dysfunction may reflect compensatory mechanisms that prevent cognitive decline [7-9].

In this Hot Topic issue of Current Neuropharmacology, we have asked notable experts in the field to comment on some of the promising directions in Alzheimer’s disease research that appear to be amenable to pharmacological intervention. In this issue, *Niedowicz *provides an insightful evaluation of AD therapeutics in light of the recent results from human clinical trials. *Martin *provides a review on the use of the aged canines as a powerful model of Alzheimer’s disease. Our experts also cover notable advances in drug targets, including the development of a unique class of high potency gamma secretase modulatory compounds (*Bulic*), the impact of calcineurin hyperactivity (*Reese*), and the pharmacological manipulation of heat shock proteins (*Dickey*) in Alzheimer’s disease. Together, we hope these reviews will provide important insights into the molecular pathways underlying the pathogenesis of this disorder, and the potential counter-measures that may serve as future therapeutic strategies for the treatment of Alzheimer’s disease.
